# Histone Acetylation Defects in Brain Precursor Cells: A Potential Pathogenic Mechanism Causing Proliferation and Differentiation Dysfunctions in Mitochondrial Aspartate-Glutamate Carrier Isoform 1 Deficiency

**DOI:** 10.3389/fncel.2021.773709

**Published:** 2022-01-12

**Authors:** Eleonora Poeta, Sabrina Petralla, Giorgia Babini, Brunaldo Renzi, Luigi Celauro, Maria Chiara Magnifico, Simona Nicole Barile, Martina Masotti, Francesca De Chirico, Francesca Massenzio, Luigi Viggiano, Luigi Palmieri, Marco Virgili, Francesco Massimo Lasorsa, Barbara Monti

**Affiliations:** ^1^Department of Pharmacy and Biotechnology, University of Bologna, Bologna, Italy; ^2^Department of Biosciences, Biotechnologies and Biopharmaceutics, University of Bari Aldo Moro, Bari, Italy; ^3^CNR Institute of Biomembranes, Bioenergetics and Molecular Biotechnologies, Bari, Italy; ^4^Department of Biology, University of Bari Aldo Moro, Bari, Italy

**Keywords:** white matter disorder, mitochondria, epigenetics, oligodendrocytes, neurons, SLC25A12/aralar1/AGC1 deficiency

## Abstract

Mitochondrial aspartate-glutamate carrier isoform 1 (AGC1) deficiency is an ultra-rare genetic disease characterized by global hypomyelination and brain atrophy, caused by mutations in the *SLC25A12* gene leading to a reduction in AGC1 activity. In both neuronal precursor cells and oligodendrocytes precursor cells (NPCs and OPCs), the AGC1 determines reduced proliferation with an accelerated differentiation of OPCs, both associated with gene expression dysregulation. Epigenetic regulation of gene expression through histone acetylation plays a crucial role in the proliferation/differentiation of both NPCs and OPCs and is modulated by mitochondrial metabolism. In AGC1 deficiency models, both OPCs and NPCs show an altered expression of transcription factors involved in the proliferation/differentiation of brain precursor cells (BPCs) as well as a reduction in histone acetylation with a parallel alteration in the expression and activity of histone acetyltransferases (HATs) and histone deacetylases (HDACs). In this study, histone acetylation dysfunctions have been dissected in *in vitro* models of AGC1 deficiency OPCs (Oli-Neu cells) and NPCs (neurospheres), in physiological conditions and following pharmacological treatments. The inhibition of HATs by curcumin arrests the proliferation of OPCs leading to their differentiation, while the inhibition of HDACs by suberanilohydroxamic acid (SAHA) has only a limited effect on proliferation, but it significantly stimulates the differentiation of OPCs. In NPCs, both treatments determine an alteration in the commitment toward glial cells. These data contribute to clarifying the molecular and epigenetic mechanisms regulating the proliferation/differentiation of OPCs and NPCs. This will help to identify potential targets for new therapeutic approaches that are able to increase the OPCs pool and to sustain their differentiation toward oligodendrocytes and to myelination/remyelination processes in AGC1 deficiency, as well as in other white matter neuropathologies.

## Introduction

Mitochondrial aspartate-glutamate carrier isoform 1 (AGC1) deficiency (DEE39; OMIM #612949; ICD-10 Code: G31.8; ORPHA Nr: ORPHA353217) is an ultra-rare (less than 10 cases known worldwide) developmental and epileptic encephalopathy caused by mutations in *SLC25A12* gene encoding the AGC1, a member of the SLC25 family of transport proteins of the inner mitochondrial membrane. Young patients develop normally during the first months of life, and subsequently begin to have seizures, muscular hypotonia, and psychomotor retardation. MRI showed decreased cerebral volume and hypomyelination with reduced content of N-acetyl aspartate (NAA), which is an acetate donor for myelin lipid synthesis (Wibom et al., 2009; Falk et al., 2014; Pfeiffer et al., 2020). More recent MRI support for the classification of SLC25A12-related disease as leukodystrophy, but this is still debated (Kavanaugh et al., 2019).

In humans, AGC1/SLC25A12 (also named, aralar1) is expressed in the brain and muscles, while the second isoform, AGC2/SLC25A13 (also named, citrin) is mainly expressed in the liver. Both the AGC isoforms catalyze the import of cytosolic glutamate plus a proton in the matrix, in exchange for aspartate, and they are components of the malate-aspartate shuttle (MAS) that allows for the entry of the glycolysis-derived NADH to the mitochondria essential for correct pyruvate oxidation (Llorente-Folch et al., 2013). Different mutations in the *SLC25A12* gene have been identified, all leading to the impairment of AGC1 activity and reduced NAA content in the patients’ brain, along with the onset of pathological features similar to other white matter diseases (Wibom et al., 2009; Falk et al., 2014; Pfeiffer et al., 2020). Neurons are the primary producers of NAA in the Central Nervous System (CNS) and they deliver this metabolite to oligodendrocytes as a source of acetyl moieties that are needed to produce myelin-associated lipids. This implies a continuous cross-talk between neurons and oligodendrocytes, with an exchange of NAA to support myelination (Moffett et al., 2007; Nave and Werner, 2014).

Until now, studies have mainly focused to mature neurons in the murine model of AGC1 deficiency, showing profound neuronal metabolic disturbances with a limited NAA production especially affecting the nigrostriatal pathway (Ramos et al., 2011; Llorente-Folch et al., 2013; Juaristi et al., 2017). More recently, it has been demonstrated that the downregulation of AGC1 inhibits proliferation and NAA synthesis in neuronal precursor cells (NPCs), as well as reduces the proliferation of oligodendrocytes precursor cells (OPCs) leading to their spontaneous and precocious differentiation in both *in vitro* and *in vivo* murine models. Interestingly, while the proliferation defects in NPCs are associated with a reduced mitochondrial respiration causing energy fault (Profilo et al., 2017), in OPCs, this appears related to a dysregulation in the expression of trophic factors and receptors involved in the proliferation/differentiation processes (Petralla et al., 2019). Since epigenetic regulation of gene expression through histone acetylation is involved in the proliferation/differentiation of both NPCs and OPCs (Juliandi et al., 2010; Emery and Lu, 2015; Hernandez and Casaccia, 2015) and NAA can act as a source of acetate for histone acetylation (Long et al., 2013; Bogner-Strauss, 2017), a reduced AGC1 activity may be linked to epigenetic/transcriptional changes affecting the OPCs pool maintenance, their differentiation toward oligodendrocytes and, therefore, lead to the myelination/remyelination processes. To test our hypothesis, in this study, we focused on the transcription factors known to be involved in the proliferation/differentiation of OPCs and NPCs, as well as on histone post-translational modifications (PTMs), histone acetyltransferases (HATs), and histone deacetylates (HDACs) in two different *in vitro* models of AGC1 deficiency: a stable clone of immortalized murine OPCs (Oli-Neu) with a partial silencing of AGC1 and the relative control and neurospheres from the sub-ventricular zone (SVZ) of AGC1^±^ and AGC1^+/+^ mice, as a model of NPCs (Petralla et al., 2019). Finally, to better clarify HATs and HDACs implication in the unbalance regulation of brain cells in the biological processes, we performed pharmacological inhibitions through the general HDAC inhibitor, suberanilohydroxamic acid (SAHA; Zhou et al., 2011), approved by FDA for cancer therapy, and the natural compound, curcumin, a specific HAT p-300 activity inhibitor (Sunagawa et al., 2018), in both the AGC1 deficiency models of siAGC1 Oli-neu cells and AGC1^±^ mice-derived neurospheres.

## Materials and Methods

### Cell Cultures

Oli-Neu cells (kindly provided by Jacqueline Trotter, University of Mainz, Germany, RRID:CVCL_IZ82) stably transfected with a scrambled control, shRNA, or an shRNA targeting the AGC1 coding sequence, which induces a 60% reduction of carrier expression, were obtained as previously published (Petralla et al., 2019). Cells were grown at 37°C and 5% CO_2_ on poly-L-lysine (10 μg/ml; Sigma-Aldrich, St Louis, MO, United States) coated Petri dishes in SATO medium [DMEM basal medium, 2 mM of glutamine, 10 μg/ml of insulin, 5.5 μg/ml of transferrin, 38.72 nM of sodium selenite, 100 μM of putrescine, 520 nM of L-thyroxine (T4), 500 nM of triiodo-L-thyronine (T3), 200 nM of progesterone, 25 μg/ml of gentamycin; all from Sigma-Aldrich, excluding insulin-transferrin-sodium selenite 100X supplement, Thermo Fisher Scientific, Waltham, Massachusetts, United States], supplemented with 1% heat-inactivated Horse Serum (HS; Sigma-Aldrich, St Louis, MO, United States) and 1 μg/ml of puromycin (Sigma-Aldrich, St Louis, MO, United States). Once confluent, the cells were detached with 0.01% trypsin–0.02% ethylenediaminetetraacetic acid (EDTA)-Hank’s Balanced Salt Solution (HBSS; Sigma-Aldrich, St Louis, MO, United States).

### Oxygen Consumption Rates Measurements

Oxygen consumption rates (OCRs) were measured with an XF^96^ Extracellular Flux analyzer (Agilent Technologies, MA, United States). The 30,000 cells/well were incubated for 1 h in a humidified incubator at 37°C in the presence of unbuffered XF base medium supplemented with 1 g/l glucose, 1 g/l glucose with 1 mM pyruvate, 1 g/l glucose with 2 mM glutamine, or 1 g/l glucose with 1 mM pyruvate and 2 mM glutamine. After incubation, OCRs were measured as previously described (Brand and Nicholls, 2011). More in the detail, basal OCRs were recorded, prior to the sequential injections of 2 μM of oligomycin, as an inhibitor of ATP synthase and indicating the oxygen consumption associated with mitochondrial ATP production (three measurements for total 15 min), 0.5 μM of FCCP, as mitochondrial uncoupler to collapse the mitochondrial membrane potential and to determine the maximal respiratory capacity (four measurements for total 15 min), and 1 μM antimycin A with 1 μM of rotenone, as mitochondrial respiratory chain inhibitors to evaluate the non-mitochondrial oxygen consumption (three measurements for total 15 min).

### Extraction and Isolation of Histones

The histone component of Oli-Neu nuclei was isolated and purified by using the acid extraction protocol (Shechter et al., 2007). The 5 × 10^6^ cells were lysed in 1 ml of hypotonic lysis buffer (10 mM of Tris-Cl pH 8.0, 1 mM of KCl, 1.5 mM of MgCl_2_, 1 mM of DTT, and 1X protease and phosphatase inhibitor cocktails; all from Sigma-Aldrich, St Louis, MO, United States). After 30 min at 4°C in mild shaking to favor hypotonic swelling and lysis, intact nuclei were pelleted 10,000 × g for 10 min at 4°C, resuspended in 400 μL of 0. N H_2_SO_4_, and incubated for 30 min in rotation. After centrifugation at 16,000 × g for10 min at 4°C, 100% of trichloroacetic acid (TCA; Sigma-Aldrich, St Louis, MO, United States) was added dropwise to the supernatant to allow histones precipitation overnight at 4°C. The following day, the solution was centrifuged at 16,000 × g for 10 min at 4°C and the pellet was washed twice in glacial acetone, dried at room temperature, and resuspended in phosphate-buffered saline (PBS) with 1X protease and phosphatase inhibitor cocktails. All samples were sonicated with a Branson 250 digital sonifier, before quantification for subsequent Western Blot (WB) analysis.

### Subcellular Fractionation

Cytosolic, mitochondrial, and nucleic extracts were obtained from a modified Grove BD and Bruckey protocol (Grove and Bruchey, 2001). Oli-Neu cells were lysed with a Potter homogenizer (B. Braun, Melsungen AG) in an isotonic buffer (10 mM of Hepes, 200 mM of mannitol, 70 mM of sucrose, 1 mM of EDTA pH 7.6, 1X protease and phosphatase inhibitor cocktails; all from Sigma-Aldrich, St Louis, MO, United States) and centrifuged at 800 × g for 10 min at 4°C. The supernatant (cytoplasmic fraction; CF) was separated from the pellet (nucleic fraction) that was washed two times with buffer A (2 mM of Hepes pH 7.9, 1 mM of NaCl, 3 mM of MgCl_2_, 0.1% of NP40, 10% of glycerol, 0.2 mM of EDTA, 1 mM of DTT, 1X protease and phosphatase inhibitor cocktails) and then with buffer B (20 mM of Hepes pH 7.9, 0.2 mM of EDTA, 200 mM of glycerol, 1 mM of DTT, 1X protease and phosphatase inhibitor cocktails). Washed nuclei were resuspended in the extraction buffer with salt (20 mM of Hepes, pH 7.9, 400 mM of NaCl, 2% of sodium dodecyl sulfate (SDS), 0.2 mM of EDTA, 20 mM of glycerol, 1 mM of DTT, 1X protease and phosphatase inhibitor cocktails). To obtain mitochondria, CF was centrifuged at 14,000 × g/20 min/4°C and the pellet (mitochondria) was resuspended in isotonic buffer with 1X protease and phosphatase inhibitor cocktails. The total protein contents were quantified (Lowry et al., 1951) and stored at −80°C until used.

### Activities of Histone Acetyltransferase and Histone Deacetylases Assay

To quantify the activities of HATs and HDACs in Oli-Neu cells, the HAT Activity Assay Kit (Abcam, Cambridge, United Kingdom) and the Epigenase HDAC Activity/Inhibition Direct Assay Kit (EpigGenetek, NY, United States) were respectively used, according to the manufacturer’s instruction. For HATs activity assays, 50 μg of nuclear extract in 40 μL of water (final volume) were added in a 96-well plate; 40 μL of water instead of samples were used for background reading; 10 μL of cell nuclear extract (NE) were added to 30 μL of water as a positive control. Depending on color development, the plates were incubated 1/4 h at 37°C and read OD440 nm at different times during incubation. To measure the activity of the HDACs, 5 μg of nuclear extracts were diluted with kit-specific reagents up to 50 μL/well (final volume). Only the reagents were used as a blank sample. The signal was detected at 450 nm with a Multiplate Spectrophotometric Reader (Bio-rad Laboratories, Milano, Italy) after 1–2 h of incubation.

### Neurospheres Preparation

Neurospheres were initially acquired from the SVZ of 8-months-old C57BL/6N wild-type and heterozygous SLC25A12 male mice (*Mus musculus*), generated by the Texas A&M Institute for Genomic Medicine (Houston, Texas, United States), as previously described (Petralla et al., 2019). Animals were fed *ad libitum* with the 2018 Teklad global diet (Envigo, United States), in a 12/12-h light-dark cycle at 20 ± 2°C and set at humidity; appropriate environmental enrichments were placed to guarantee their well-being. All animal experiments were authorized by a local bioethical committee (Protocol no 3/79/2014) and performed in agreement with the Italian and European Community law (Directive 2010/63/EU) on the use of animals for experimental purposes, and adherence to the ARRIVE Reporting Guidelines. Neurospheres were obtained through SVZ microdissection on three AGC1^+/+^ and three AGC1^±^ mice, respectively, previously anesthetized through intraperitoneal injection of 10 mg/kg xylazine followed by cervical dislocation. Fresh tissue was mechanically dissociated in HBSS, 3.9 mg/ml of N-2-hydroxyethylpiperazine-N-2-ethane sulfonic acid (HEPES), 0.5 mg/ml of NaHCO_3_, 0.9 mg/ml of glucose, 0.5% penicillin/streptomycin, and centrifuged for 5 min at 1,000 rpm. The pellet was resuspended in papain solution (0.2 mg/ml of EDTA, 0.66 mg/ml of Papain, 0.2 mg/ml of cysteine in HBSS) and placed for 20 min at 37°C shaking at every 5 min. For further dissociation, the tissue was resuspended in HBSS and left for another 10 min at 3°C. Papain reaction was then inhibited by adding DMEM F-12 (Gibco Life Technologies, Waltham, MA, United States), and samples were centrifuged at 100 rpm for 5 min. The cells were plated in 35 mm dishes in a complete culture medium: DMEM-F12 (Gibco Life Technologies, Waltham, MA, United States) was supplemented with 2 mM of glutamine, 10 μg/ml of insulin from bovine pancreas (Sigma-Aldrich, St Louis, MO, United States), 20 ng/ml of epidermal growth factor (EGF; PeproTech EC, London, United Kingdom), 20 ng/ml of fibroblast growth factor-2 (FGF2; PeproTech), 1% of N2 (Thermo Fisher Scientific, Waltham, MA, United States), 1% of B27 (Thermo Fisher Scientific, Waltham, MA, United States), 10 units/ml of penicillin and 10 μg of streptomycin. Neo-formed neurospheres were cultured and passed every week (5/7 days of growth). For this purpose, the cells were collected and pelleted for 5 min at 1,000 rpm, washed in PBS, and centrifuged again for 5 min at 1,000 rpm. The neurospheres were dissociated through 5 min incubation in Accutase (Aurogene Srl, Roma, Italy) at 37°C, and basal DMEM F-12 was added to stop the reaction. Following centrifugation for 5 min at 1,000 rpm, single cells were resuspended in a complete culture medium to proceed with cell count in order to obtain a final cell density of 5 × 10^3^ cells/cm^2^ in 35 mm dishes. After passage 3, the neurospheres were stabilized as stable clones to be used for the experiments.

### Curcumin and Suberanilohydroxamic Acid Treatments

To act on HATs and HDACs activity, Oli-Neu cells and the neurospheres were treated with the specific HAT p300 inhibitor, curcumin (Sunagawa et al., 2018) and the general HDAC inhibitor, SAHA (Zhou et al., 2011) that has been approved by FDA for cancer therapy, respectively. For WB and microscopy analysis on Oli-Neu cells, 2 × 10^5^ cells/well were plated in a 6-well plate or 24 mm diameter glass coverslips, both previously treated with poly-L-lysine (10 μg/ml). After 2 h, complete SATO medium was replaced with a fresh medium containing SAHA (0.5 μM; 1 μM) or curcumin (10 μM; 20 μM), and the cells were incubated at 37°C in 5% CO_2_ for 24 or 48 h depending on the proliferation or differentiation assays. The same dimethyl sulfoxide (DMSO) volume that was required to dissolve the molecules was used as a control. For immunostainings, the cells on glass coverslips were then fixed with 4% of paraformaldehyde (PFA) in PBS 0.1% pH 7.4 for 30 min, washed with PBS, and stored at 4°C in PBS. For WB analysis, the Oli-Neu on dishes were collected with the lysis buffer (50 mM of Tris pH7.4, 1% SDS, 1 mM of EDTA, 1X protease and phosphatase inhibitor cocktails) and kept at −80°C until use. In parallel, to study HATs and HDACs inhibition on neurospheres proliferation, the cells were plated as single stem cells in 96-well plates (5 × 10^3^cells/well) in presence of SAHA (0.5 μM; 1 μM) or curcumin (5 μM; 10 μM) in complete DMEM F-12 culture medium; same DMSO volumes were used as control. Depending on the inhibitor’s toxicity after a long-time in culture, the neurospheres were let grown 5 days in the presence of SAHA and 7 days with curcumin. In contrast, to evaluate the differentiation of neurospheres, 75 or 30 spheres were plated on 35 mm Petri dishes or 13 mm glass coverslips in complete DMEM F-12 medium and inhibitors, for subsequent WB or immunofluorescence analysis, respectively. To allow for stem cell adhesion and neuronal differentiation, both the dishes and coverslips previously treated with poly-L-lysine (10 μg/ml) were incubated at 37°C with fibronectin (1 μg/ml) for at least 3 h. After 7 days in culture, the differentiated neurospheres were collected in a lysis buffer and stored at −80°C, or fixed for 30 min with 4% of PFA in PBS 0.1% pH 7.4 and kept at 4°C in PBS.

### Western Blot

The samples of oli-Neu cells and neurospheres in lysis buffer (50 mM Tris pH 7.4, 1% of SDS, 1 mM of EDTA, 1X protease and phosphatase inhibitor cocktails), were sonicated with a Branson 250 sonifier. Each sample (20 μg) was resolved in SDS–PAGE with Laemli loading buffer (Sigma-Aldrich, St Louis, MO, United States) and transferred onto a nitrocellulose membrane (GE Healthcare Life Sciences, Little Chalfont, United Kingdom) for the reaction with the following primary antibodies: Anti-acetyl-Histone H3 (Millipore, Burlington, Massachusetts, United States; Cat# 06-599, RRID:AB_2115283), CBP (D6C5) (Cell Signaling Technology, Danvers, Massachusetts; Cat# 7389, RRID:AB_2616020), CNPase (Cell Signaling; Cat# 5664, RRID:AB_10705455), CREB (48H2) (Cell Signaling; Cat# 9197, RRID:AB_331277), c-Myc (N-262) (SantaCruz Biotechnology, Dallas, Texas, United States; Cat# sc-764, RRID:AB_631276), Anti-Doublecortin (Abcam; Cat# ab18723, RRID:AB_732011), GAPDH (SantaCruz Biotechnology, Dallas, Texas, United States; Cat# sc-32233, RRID:AB_627679), GFAP (Dakopatts (Agilent Technologies), Santa Clara, California, United States; Cat# sc-33673, RRID:AB_627673), Histone Deacetylase 1 (HDAC1) (Cell Signaling; Cat# 2062, RRID:AB_2118523), HDAC2 (D6S5P) (Cell Signaling; Cat# 2540, RRID:AB_2116822), Histone Deacetylase 3 (HDAC3) (Cell Signaling; Cat# 2632, RRID:AB_331545), Histone Deacetylase 4 (HDAC4) (Cell Signaling; Cat# 2072, RRID:AB_2232915), Histone H3 (SantaCruz Biotechnology, Dallas, Texas, United States,; Cat# sc-10809, RRID:AB_2115276), HSP60 (Bioss, Woburn, Massachusetts, United States; Cat# bs-0191R-HRP, RRID:AB_11117391), MAX (H-2) (SantaCruz Biotechnology, Dallas, Texas, United States; Cat# sc-8011, RRID:AB_627913), Anti-NG2 (Abcam, Cambridge, United Kingdom; Cat# ab83178, RRID:AB_10672215), NRSF (P-18) (SantaCruz Biotechnology, Dallas, Texas, United States; Cat# sc-15120, RRID:AB_2179628), Olig2 (SantaCruz Biotechnology, Dallas, Texas, United States; Cat# sc-48817, RRID:AB_2157550), PanMetH3 (MBL International, Woburn, Massachusetts, United States; Cat# LS-A4069, RRID:AB_591306), PDGFRα (SantaCruz Biotechnology, Dallas, Texas, United States; Cat# sc-338, RRID:AB_631064), Phospho-CREB (Ser133) (Cell Signaling; Cat# 9198, RRID:AB_2561044). The following specific HRP-linked secondary antibodies (horseradish peroxidase conjugated) were used: Goat anti-Mouse (Jackson ImmunoResearch, West Grove, Pennsylvania, United States; Cat# 115-035-146, RRID:AB_2307392), Goat anti-Rabbit (Jackson ImmunoResearch; Cat# 111-035-144, RRID:AB_230739), and Mouse anti-Goat (SantaCruz Biotechnology, Dallas, Texas, United States; Cat# sc-2354, RRID:AB_628490). Labeled proteins were then detected by using Clarity™ Western ECL Substrate (Bio-Rad). Densitometric analysis were performed by using Biorad Image Lab software 6.0.0 (RRID:SCR_014210). All primary antibodies were diluted 1:1,000 excl. GAPDH 1:20,000, whereas secondary antibodies were diluted at 1:5,000 in 0.1% Tween-20/PBS.

### Immunofluorescence Analysis

Oli-Neu cells and neurospheres fixed on coverslips were permeabilized in 0.1% Triton X-100/PBS and then incubated with the following primary antibodies: AGC1/Aralar1 (SantaCruz Biotechnology, Dallas, Texas, United States; Cat# sc-271056, RRID:AB_10608837), CBP (D6C5) (Cell Signaling T; Cat# 7389, RRID:AB_2616020), CNPase (Cell Signaling; Cat# 5664, RRID:AB_10705455), c-Myc (9E10) (SantaCruz Biotechnology, Dallas, Texas, United States; Cat# sc-764, RRID:AB_631276), Anti-Doublecortin (Abcam; Cat# ab18723, RRID:AB_732011), GFAP (Dakopatts; Cat# sc-33673, RRID:AB_627673), HDAC2 (D6S5P) (Cell Signaling; Cat# 2540, RRID:AB_2116822), Histone Deacetylase 3 (HDAC3) (Cell Signaling; Cat# 2632, RRID:AB_331545), HSP60 (Bioss; Cat# bs-0191R-HRP, RRID:AB_11117391), anti-Ki67 (Abcam Cambridge, United Kingdom; Cat# ab15580, RRID:AB_443209), MAX (H-2) (SantaCruz Biotechnology, Dallas, Texas, United States; Cat# sc-8011, RRID:AB_627913), NRSF (P-18) (SantaCruz; Cat# sc-15120, RRID:AB_2179628), Olig2 (SantaCruz Biotechnology, Dallas, Texas, United States; Cat# sc-48817, RRID:AB_2157550), and Phospho-CREB (Ser133) (Cell Signaling; Cat# 9198, RRID:AB_2561044). The fluorescent secondary antibodies used were as follows: Donkey anti-Mouse IgG Alexafluor 555 (Abcam, Cambridge, United Kingdom; Cat# ab150106, RRID:AB_2857373), Goat anti-Mouse IgG Alexafluor 488 (Abcam Cambridge, United Kingdom; Cat# ab150113, RRID:AB_2576208), Goat anti-Rabbit IgG Alexafluor 488 (Abcam, Cambridge, United Kingdom; Cat# ab150077, RRID:AB_2630356), and Goat anti-Rabbit IgG Alexafluor 555 (Abcam; Cat# ab150078, RRID:AB_2722519). Nuclei were stained with Hoechst 33258 (Sigma-Aldrich) or DAPI (Santa Cruz Biotechnology, Dallas, Texas, United States, Cat#. sc-24941). For cell counting, stained Oli-Neu cells, three randomly selected fields/coverslip were acquired by using the Nikon EZ-C1 microscope (10X or 100X objective); positive cells were counted and the labeling index was expressed as the ratio of positive/total cells using Fiji software (ImageJ2, Fiji; RRID:SCR_002285). The confocal images of the neurospheres were obtained with 10 X or 60 X objective and the z-stack function (40 total stacks), and 3D image reconstruction were performed by using Fiji ImageJ2 software z-project plugin. The fluorescence intensity index was estimated as the ratio of the markers’ positive cells intensity/total cells fluorescence intensity stained with DAPI.

### Neurospheres Proliferation

To evaluate the growth rate of AGC1^+/+^ and AGC1^±^ neurospheres, following HATs and HDACs inhibition, five different images were acquired in bright field mode (10 X objective) for each 96-well (5 × 10^3^ cells/well) by using an Eclipse TE2000-s—Nikon microscope. The images were analyzed with Fiji ImageJ2 using the publicly available colony and cell counting method (Choudhry, 2016) and only aggregates with areas greater than 400 μm^2^ were considered.

### Oli-Neu Cells Extensions Number and Length Measurement

To analyze the extension number and length of Oli-Neu cells in the presence or absence of inhibitors, five randomly selected fields for each 6-well (2 × 10^5^ cells/well) were acquired (20 × objective) with an Eclipse TS100—Nikon microscope. The length of the processes was measured with Fiji ImageJ2 software. Scale bar distance in pixels and the corresponding distance in micrometers were set by using the reference scale bar and the SET SCALE function (Analyze menu). Each cell process was traced with the segmented-line function and the MEASURE function (Analyze menu) was used to determine the length of the extensions in micrometers. The number of processes was directly determined from individual processes of length measurement.

### Statistical Analysis

All results were subjected to statistical analysis by using Student’s *t*-test or one-way ANOVA followed by Bonferroni’s *post hoc* comparison test. In drug treatments, to evaluate both AGC1 silencing and HATs/HDACs inhibition, two-way ANOVA followed by Dunnett’s *post hoc* comparison test, was used. Statistical analysis was performed with GraphPad Prism 4 software (GraphPad Prism, San Diego, CA, United States; RRID:SCR_002798), and only *p-*values < 0.05 were considered statistically significant.

## Results

### Expression of Transcription Factors in Aspartate-Glutamate Carrier Isoform 1-Deficient Oligodendrocytes Precursor Cells and Neuronal Precursor Cells

**FIGURE 1:**
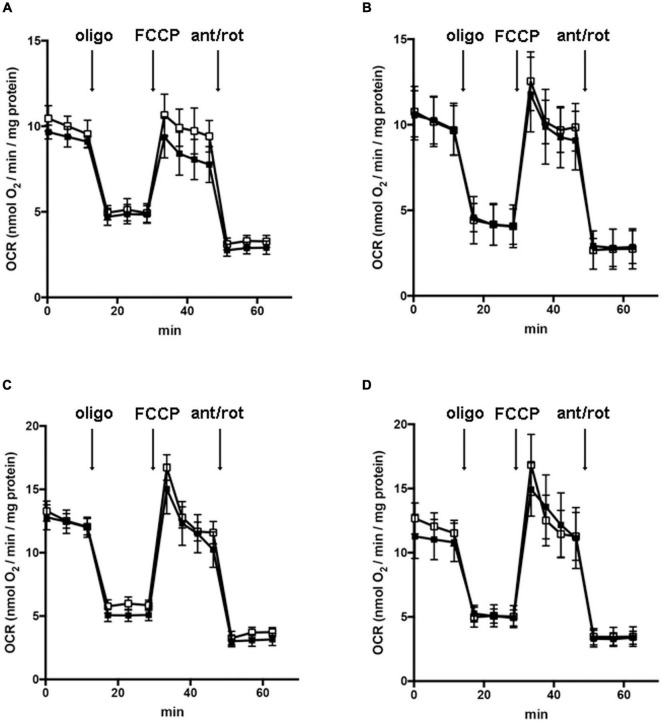
Downregulation of AGC1 does not inhibit mitochondrial respiration in Oli-Neu cells. Oxygen consumption rates (OCRs) were measured with XF^96^ extracellular flux analyzer (SeaHorse; Agilent Technologies, MA, United States) in control (□) and siAGC1 Oli-Neu (■) cells incubated for 1 h in XF base medium supplemented with 1 g/l glucose **(A)**, 1 g/l glucose with 1 mM pyruvate **(B)**, 1 g/l glucose with 2 mM glutamine **(C)**, or 1 g/l glucose with 1 mM pyruvate with 2 mM glutamine **(D)**. Oli-Neu cells were exposed to sequential additions of 2 μM oligomycin, 0.5 μM FCCP, and 1 μM antimycin A with 1 μM rotenone. OCR data were normalized to cell protein content. Mean values ± SD from three independent experiments each including 5–6 replicates per cell type are shown.

**FIGURE 2:**
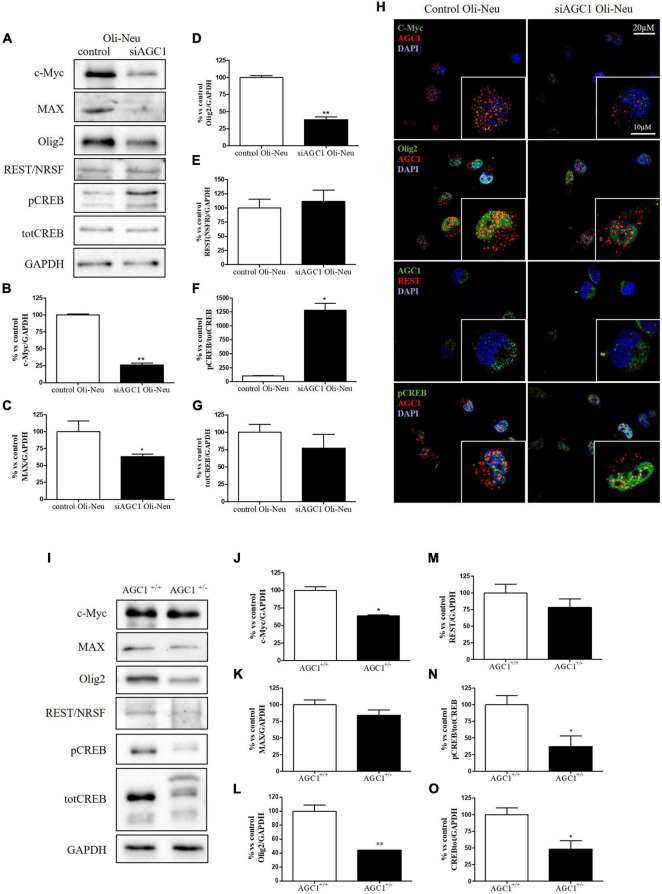
Different expression of proliferation/differentiation transcription factors characterizes both Oli-Neu and neurospheres AGC1 deficiency *in vitro* models. WB and relative densitometries of c-Myc **(A,B)**, MAX **(A,C)**, Olig2 **(A,D)**, REST **(A,E)**, pCREB **(A,F)**, and total CREB **(A,G)** expression in Oli-Neu cells; GAPDH was used for endogenous normalization. Confocal microscopy images (100X) of the transcription factors **(H)** in Oli-Neu cells; nuclei were labeled with DAPI. Scale bars: 20 and 10 μm. WB analysis and relative densitometries of c-Myc **(I,J)**, MAX **(I,K)**, Olig2 **(I,L)**, REST **(I,M)**, pCREB **(I,N)**, and total CREB **(I,O)** expression in neurospheres; GAPDH was used for endogenous normalization. Values are mean ± SD of at least 3 independent experiments; **p < 0.01, *p < 0.05, compared to controls; Student’s *t*-test.

In order to understand whether the dysregulation in trophic factors and receptors involved in the proliferation deficit of OPCs with AGC1 deficiency could be related to a transcriptional alteration, we evaluated the expression of transcription factors that are known to play a role in the proliferation/differentiation of BPCs, such as c-Myc (Magri et al., 2014; Palazuelos et al., 2014) and its co-factor, Max (Carroll et al., 2018), Olig2 (Meijer et al., 2012), the Repressor element 1 silencing transcription factor/neuron-restrictive silencing factor (REST/NRSF) (Song et al., 2015), as well as the cAMP response element binding protein (CREB) (Yan et al., 2013). We initially performed WB analysis on Oli-Neu (immortalized mouse OPCs) cells, with a stable downregulation of the *SLC25A12* gene (*siAGC1*) and on control cells, as well on the NPCs model of neurospheres, derived from the SVZ of C57BL/6N wild-type (AGC1^+/+^) and AGC1^±^ mice, both generated as previously described (Petralla et al., 2019). Oxygen consumption rate (OCR) measurements confirmed that siAGC1 Oli-Neu cells do not exhibit a mitochondrial respiratory deficit compared to the control cells (Figure 1). However, siAGC1 Oli-Neu cells revealed an altered transcriptional profile with a significant reduction in both c-Myc and Max, as well as in Olig2 expression, together with a marked increase in CREB activation through its phosphorylation on serine 133. Differently, no significant changes in total CREB and REST/NRSF were observed (Figures 2A–G). Similar data were obtained in immunofluorescence experiments with double staining for AGC1 and transcription factors. As shown in Figure 2H, we detected lower c-Myc and Olig2 labeling in siAGC1 Oli-Neu cells compared to the control cells, with no relevant differences in REST intensity, whereas phospho-CREB staining resulted increased, thus indicating an altered transcriptional profile when AGC1 is downregulated. Parallel experiments have been carried on neurospheres from AGC1^+/+^ and AGC1^±^ mice used as a near-perfect *in vitro* model to provide a consistent and self-renewable source of NSPs, which can lead to neuronal-restricted precursor cells, OPCs, and astrocytes (Tropepe et al., 1999; Petralla et al., 2019). We previously demonstrated that neurospheres from AGC1^±^ mice display a reduction in OPCs with a parallel increase in oligodendrocytes, neuronal-restricted progenitors, and astrocytes compared to those from AGC1^+/+^ animals (Petralla et al., 2019). In this study, in AGC1^±^ mice neurospheres, we found a reduction in c-Myc, but not in its co-factor Max, and a decrease in Olig2 expression. Instead, no changes were observed in total CREB and REST/NRSF, whereas CREB phosphorylation turned out the significant reduction in AGC1^±^ neurospheres compared to AGC1^+/+^ (Figures 2I–O).

Taken together, these data pointed out that, in both the models with reduced AGC1 levels, the expression pattern of transcription factors involved in the proliferation/differentiation of BPCs is altered. Olig2 and c-Myc were strongly downregulated, whereas pCREB increases in siAGC1 Oli-Neu cells and decreases in AGC1^±^ neurospheres, probably as a consequence of t more heterogeneous cellular composition of the NSCs model.

### Histone Acetylation, Histone Deacetylases, and Histone Acetyltransferases in Aspartate-Glutamate Carrier Isoform 1-Deficient Oligodendrocytes Precursor Cells and Neuronal Precursor Cells

**FIGURE 3:**
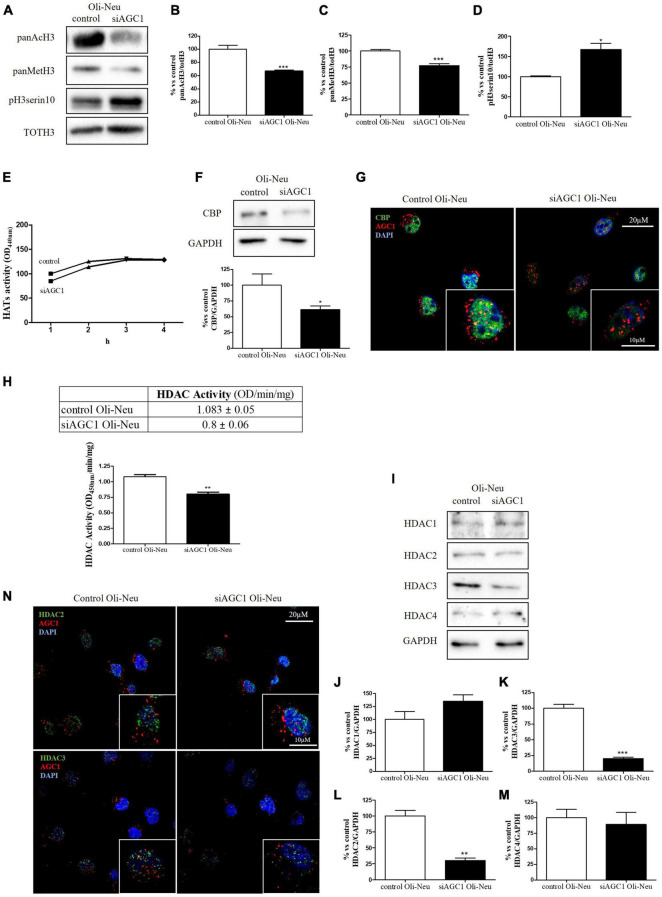
Altered histone PTMs, HAT CBP, and HDAC isoforms expression/activities in AGC1-silenced Oli-Neu cells. WB analysis and relative densitometries of panAcH3 **(A,B)**, panMetH3 **(A,C)**, pH3 **(A,D)**, CBP **(F)**, HDAC1 **(I,J)**, HDAC2 **(I,K)**, HDAC3 **(I,L)** and HDAC4 **(I,M)** expression in Oli-Neu cells; GAPDH was used for endogenous normalization. Activity assay of CBP **(E)** and HDAC **(H)** in Oli-Neu cells. Histone deacetylases (HDACs) activity mean ± SEM of control Oli-neu: 1.083 ± 0.0535, Mean ± SEM of siAGC1 Oli-neu: 0.8000 ± 0.0608, *N* = 3. Difference between means 0.2830 ± 0.04676. Confocal microscopy images (100X) of CBP **(G)** and HDAC2 and HDAC3 **(N)** (green) and AGC1 (red) in Oli-Neu cells; nuclei were labeled with DAPI. 20 and 10 μm scale bar. Values are mean ± SD of at least 3 independent experiments; ***p < 0.001, **p < 0.01, *p < 0.05, compared to control; Student’s *t*-test.

It is well known that epigenetic mechanisms related to histone modification may have an impact on the proliferation/differentiation of OPCs and NPCs (Tiane et al., 2019; Zhang et al., 2020). Therefore, in OPCs and NPCs with AGC1 deficiency, we evaluated histone PTMs, as well as the activity and the expression profiles of the two classes of enzymes promoting histone acetylation, i.e., HATs, or deacetylation processes, i.e., HDACs. From WB and immunofluorescence analysis, siAGC1 Oli-Neu cells displayed a significant reduction in both histone H3 acetylation and methylation, with a parallel increase in its phosphorylation (Figures 3A–D). In siAGC1 Oli-Neu cells, the resultant HATs activity was not significantly lower than the control cells (Figure 3E), whereas the p300/CREB-Binding Protein (CBP), the most important protein of HATs family for brain development (Sheikh, 2014; Lipinski et al., 2019), came out significantly downregulated (Figures 3F,G). Differently, the total HDACs activity proved to be significantly reduced in siAGC1 Oli-Neu cells compared to the controls (Figure 3H). Therefore, we further investigated the expression of the HDACs isoforms mainly involved in CNS development, i.e., the nuclear isoform 1, 2, and 3 of class I HDAC and the isoform 4 of the class II HDAC (Morris and Monteggia, 2013; D’Mello, 2020; Figures 3I–N). The WB analysis demonstrated that HDAC2 (Figure 3L) and HDAC3 (Figure 3K) levels were significantly lower in siAGC1 Oli-Neu cells compared to the controls, whereas HDAC1 (Figure 3J) and HDAC4 (Figure 3M) expression remained unchanged. Similar data were obtained in immunofluorescence experiments, where both HDAC2 and HDAC3 costained with AGC1 turned out to be weaker in cells where AGC1 is downregulated (Figure 3N), suggesting that AGC1 inhibition affects the balance between acetylation and deacetylation pathways in OPCs.

**FIGURE 4:**
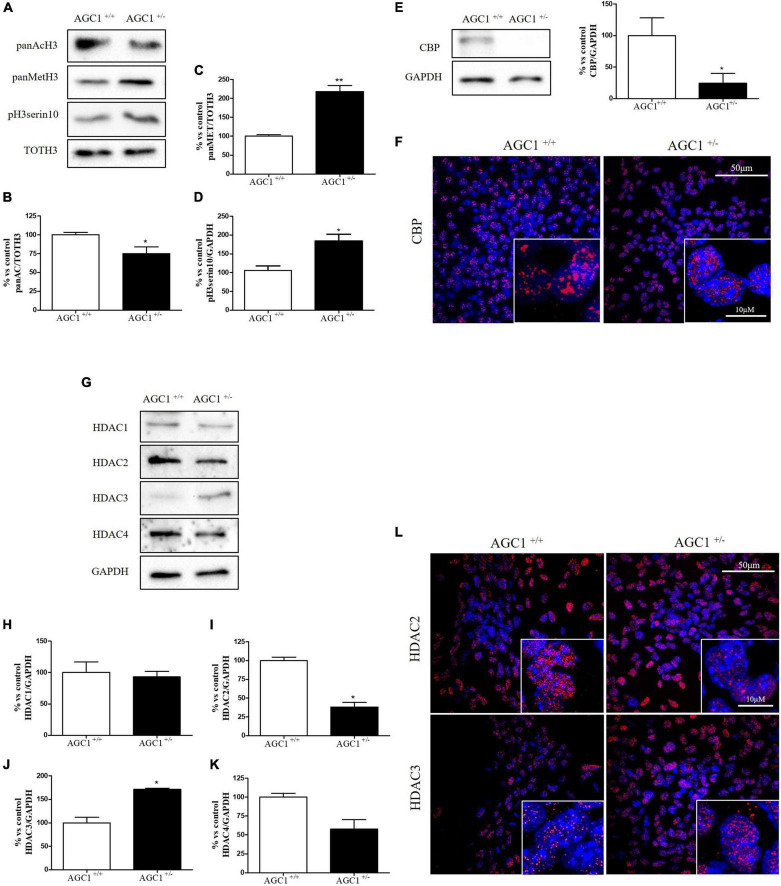
Histone H3 PTMs, HAT, and HDACs expression in AGC1^+/+^ and AGC1^±^ neurospheres. WB analysis and relative densitometries of panAcH3 **(A,B)**, panMetH3 **(A,C)**, pH3 **(A,D)**, CBP **(E)**, HDAC1 **(G,H)**, HDAC2 **(G,I)**, HDAC3 **(G,J)**, and HDAC4 **(G,K)** expression in AGC1^±^ and AGC1^+/+^ spontaneously differentiated neurospheres; GAPDH was used for endogenous normalizations. Confocal microscopy images (60X and 100X) of CBP **(F)**, HDAC2, and HDAC3 **(L)** in neurospheres; nuclei were labeled with DAPI. 50 and 10 μm scale bar. Values are mean ± SD of at least 3 independent experiments; **p < 0.01, *p < 0.05, compared to control; Student’s *t*-test.

Histone acetylation and the expression of the related enzymes were also investigated in neurospheres derived from AGC1^±^ and AGC1^+/+^ mice (Figure 4). In AGC1^±^ neurospheres, we observed lower levels of histone H3 acetylation (Figures 4A,B) vs. higher levels of histone H3 methylation (Figures 4A,C) and phosphorylation (Figures 4A,D), while CBP turned out strongly downregulated (Figures 4E,F).

**FIGURE 5:**
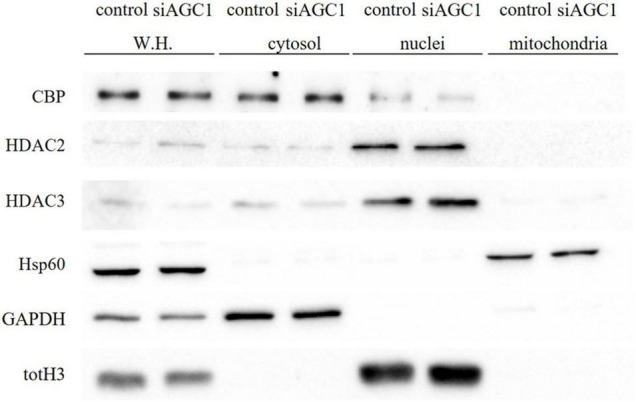
Proliferation factor c-Myc, acetyltransferase CBP and HDAC isoform 2 and 3 do not show alterations in the subcellular localization in siAGC1 Oli-Neu cells. WB analysis of c-Myc, HAT–CBP and HDAC2 and HDAC3 expression in the whole homogenate (WH), cytosolic, nuclear, and mitochondrial fractions from siAGC1 and control Oli-Neu cells; HSP60, GAPDH, and totH3 were used as mitochondrial, cytosolic, and nuclear specific markers, respectively.

Concerning HDAC isoforms, HDAC2 (Figures 4G,I), which regulates together with HDAC1 (Figures 4G,H) the oligodendorcyte and astrocytes lineage fate switch (Ye et al., 2009), and HDAC4 (Figures 4G,K) resulted downregulated in AGC1^±^ neurospheres compared to AGC1^+/+^, whereas HDAC3 (Figures 4G,J) revealed an opposite expression profile. Data were confirmed through immunofluorescence analysis (Figure 4L), where AGC1^±^ neurospheres showed lower HDAC2 labeling and higher levels of HDAC3 compared to AGC1^+/+^. Additionally, after subcellular fractionation on Oli-neu cells, no relevant differences were observed for HDAC2 and HDAC3 localization in siAGC1 cells compared to the control, whereas CBP appeared mainly localized in the cytosolic compartment and less represented in the nuclear fraction, probably as part of other different multimeric complexes (Figure 5).

Overall, these data revealed that histone PTMs are affected by AGC1 downregulation in both immortalized OPCs and NPCs, in which acetylation appeared reduced with significantly low levels of the HAT CBP. In addition, although HDACs pathways turned out differently impaired in the two distinct models, our data suggest an altered balance of HATs/HDACs expression and activity following AGC1 inhibition.

### Histone Acetyltransferases Inhibition Through Curcumin in the Proliferation/Differentiation of Oligodendrocytes Precursor Cells and Neuronal Precursor Cells

In order to clarify the role of histone acetylation in the proliferation/differentiation OPCs and NPCs, and how this biological process is involved in AGC1-deficiency brain cells proliferation defects, we further performed a pharmacological inhibition of either HATs or HDACs in both the control and siAGC1 Oli-Neu cells, as well as AGC1^+/+^ and AGC1^±^ neurospheres.

**FIGURE 6:**
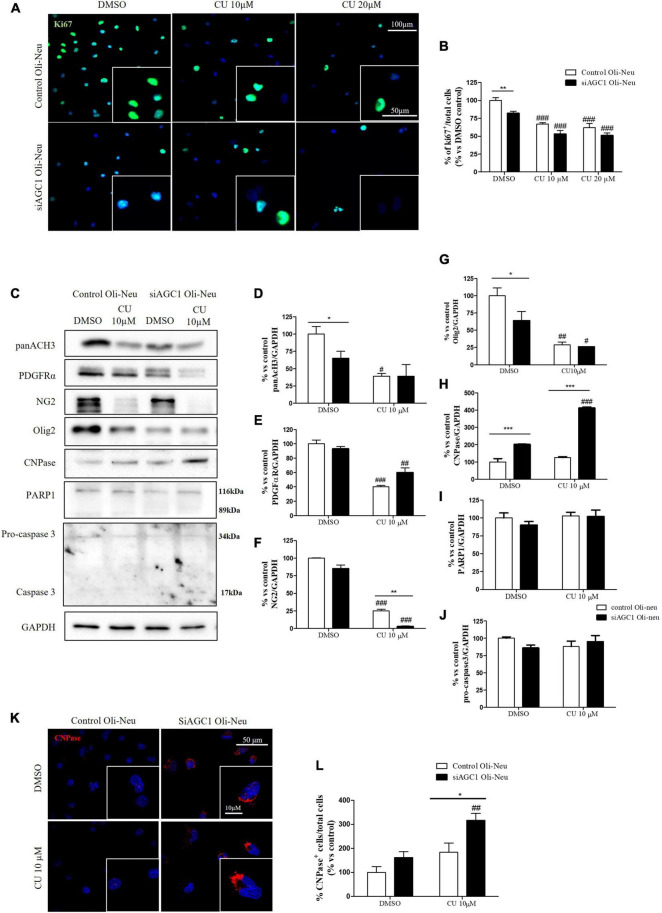
Curcumin-mediated HAT inhibition differentially affects proliferation in both control and siAGC1 Oli-Neu cells. Immunofluorescence staining of Ki67 proliferation marker **(A)** and Ki67^+^ cell count analysis **(B)** in Oli-Neu cells after 24 h treatment with 10 or 20 μM curcumin. Values are expressed as the *ratio* of Ki67^+^ cells (green)/total cells; nuclei were labeled with DAPI. Values are mean ± SD of 3 independent experiments; 3 different fields were acquired for each condition. 40X objective; 50 and 100 μm bar scale. WB and relative densitometries of panAcH3 **(C,D)**, PDGFRα **(C,E)**, NG2 **(C,F)**, Olig2 **(C,G)**, CNPase **(C,H)**, PARP1 **(C,I)**, and pro-caspase3 **(C,J)** expression in OliNeu cells after 48 h treatment with 10 μM curcumin. Immunofluorescence staining of CNPase **(K)** and CNPase^+^ cell count analysis **(L)** in Oli-Neu cells after 48 h treatment with 10 μM curcumin. Values are expressed as the *ratio* of CNPase^+^ cells (red)/total cells; nuclei were labeled with DAPI. Three different fields were acquired for each condition. Values are mean ± D of at least 3 independent experiments. 40X objective; 50 and 10 μm bar scale. ^#^*p* < 0.05, ^##^*p* < 0.01, ^###^*p* < 0.001, compared to DMSO control, respectively; *p < 0.05, **p < 0.01, ***p < 0.001, compared to each treated control; two-way ANOVA (Bonferroni’s *post hoc* test).

**FIGURE 7:**
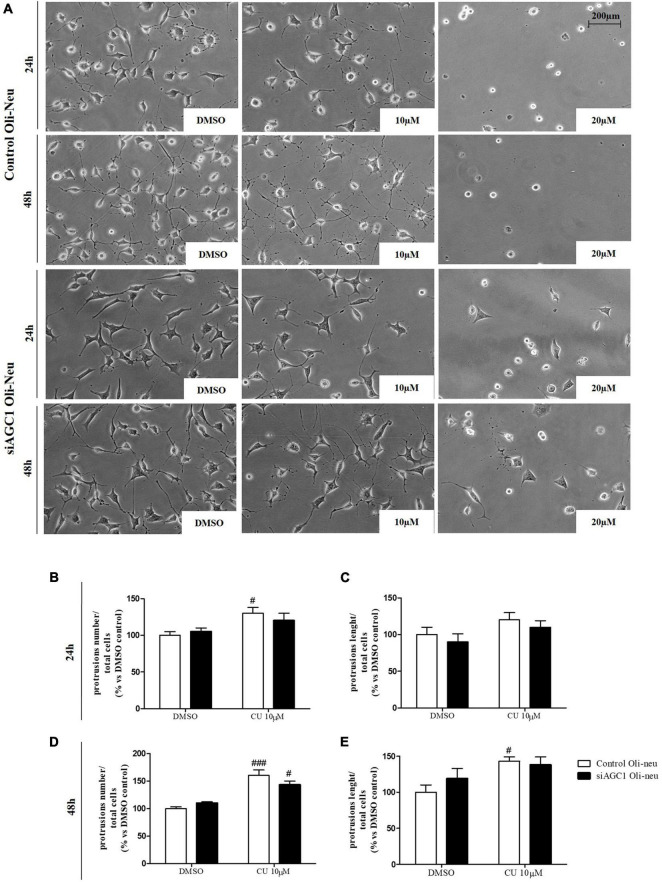
After acetyltransferase CBP inhibition, cells appeared more elongated and branched, index of oligodendrocytes maturation, showing an increase in both processes number and average length compared to DMSO-treated controls. Optical microscope proliferation/differentiation analysis in control and siAGC1 Oli-Neu cells following 24 and 48 h of treatment with curcumin 10 and 20 μM **(A)**. Filaments number and lengths were counted and measured, respectively, and analyses were carried out with Fuji Imagej2 software. Given the potential toxicity of curcumin 20 μM, statistical analyses were executed only at 10 μM **(B–E)**. Values are mean ± SD of 3 independent experiments; 3 different fields were acquired for each condition. 20X objective; 200 μM bar scale. ^###^*p* < 0.001, ^#^*p* < 0.05, compared to DMSO control, respectively; two-way ANOVA (Bonferroni’s *post hoc* test).

To inhibit HATs activity, we used curcumin, a well-known inhibitor of p300/CBP (Balasubramanyam et al., 2004). In cells, it promotes proteasome-dependent degradation of p300 and the closely related CBP protein without affecting the HATs PCAF or GCN5, in addition to inhibiting the acetyltransferase activity of purified p300 as assessed using either histone H3 or p53 as a substrate. Radiolabeled curcumin formed a covalent association with p300, and tetrahydrocurcumin displayed no p300 inhibitory activity, consistent with a Michael reaction-dependent mechanism (Marcu et al., 2006). We first evaluated the curcumin effect on Oli-Neu cells. Cell counting after staining for the proliferation marker, Ki67 (Gerdes et al., 1991; Figures 6A,B) confirmed the lower proliferation rate of siAGC1 Oli-Neu compared to control cells (DMSO treated cells) (Petralla et al., 2019) and demonstrated that 24 h curcumin treatment (used at 10 and 20 μM on the basis of preliminary did not show MTT cytotoxicity assays) induced a global reduction in proliferation levels, leading to control cells similar to Oli-Neu cells with AGC1 downregulation. This effect seemed related to HATs inhibition, as curcumin significantly reduced histone H3 acetylation in both control and siAGC1 Oli-Neu cells (Figures 6C,D). To better investigate the effect of HATs inhibition on the proliferation/differentiation of OPCs, several markers have been further evaluated after 48 h of curcumin treatment. In particular, platelet-derived growth factor receptor α (PDGFRα; Figures 6C,E), a marker of pre-progenitors and oligodendrocytes precursors (Nishiyama et al., 2021b), and the oligodendrocyte-specific transcription factor, Olig2 (Figures 6C,G), whose expression decreases during differentiation (Grinspan, 2002), appeared significantly less expressed in the control and siAGC1 Oli-Neu cells treated with curcumin compared to DMSO cells, with no more significant differences among the treated cells. In addition, curcumin significantly decreased the level of neuron glial antigen 2 (NG2; Polito and Reynolds, 2005) in the control and siAGC1 Oli-neu, to suggest a reduction in OPCs pool compared to untreated controls (Figures 6C,F). In order to exclude apoptosis and cell death, we then verified through WB analysis, the expression of the pro-apoptotic marker, caspase3 (both precursor and cleaved versions of the enzyme) (Widmann, 2007), and no differences were observed in treated vs. control cells (Figures 6C,J). Additionally, we performed WB for the full length PARP1 (116 kDa) and the large fragment (89 kDa) of PARP1 resulting from caspase cleavage (Mashimo et al., 2021; Figures 6C,I), as well as for the anti-apoptotic protein, BCL-2 (Chipuk et al., 2010) (data not shown). In parallel, in Oli-Neu cells with AGC1 downregulation, the marker of mature oligodendrocytes CNPase (Scherer et al., 1994) showed higher expression as compared to the control cells and was further induced by curcumin treatment (Figures 6C,H). This CNPase over-expression was then confirmed by immunofluorescence and cell counting (Figures 6K,L). To deepen the possible induction of differentiation by curcumin, the Oli-Neu cells were also evaluated through the protrusion number and length counting after microscopic morphology analysis. Curcumin 10 μM induced a significant increase in protrusions number both after 24 h (Figures 7A,B) and 48 h (Figures 7A,D) in control Oli-Neu cells, whereas relevant effects were observed only at 48 h in siAGC1 cells (Figures 7A,D). Differently, protrusions length significantly increased only in the control Oli-Neu cells after 48 h of treatment (Figures 7A,E). Thus, these data demonstrate that the lower AGC1 expression in immortalized OPCs determines a reduction in histone acetylation probably related to the reduction in CBP expression, as previously shown. Additionally, the pharmacological inhibition of HATs through curcumin affects proliferation, especially in the control Oli-Neu cells, leading to proliferation levels comparable to siAGC1 Oli-Neu.

**FIGURE 8:**
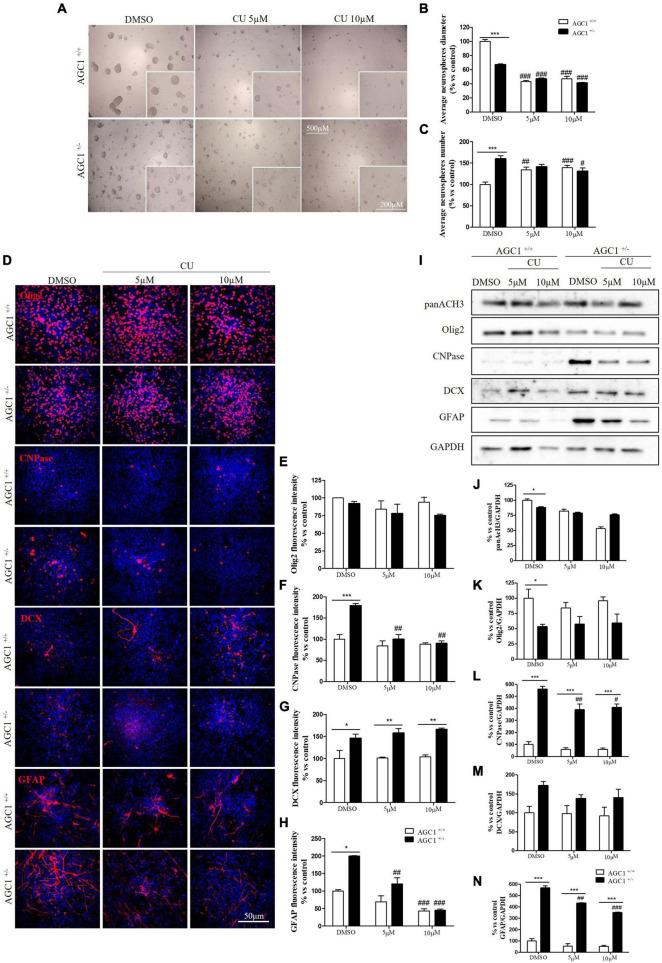
Curcumin-mediated HAT inhibition differentially affects proliferation and glial commitment in spontaneously differentiated AGC1^+/+^ and AGC1^±^ neurospheres. Optical microscope images **(A)** and counting of number **(B)**, and diameter **(C)** of neurospheres after 7 days of culture in presence of curcumin 5 and 10 μM. Average number and size were measured with Fuji Imagej2 software using an automated colony and cell counting method; only aggregates bigger than 400 μm^2^ were considered. Values are mean ± SD of 5 different fields acquired for each condition. 10X objective; 500 and 200 μm bar scale. Immunofluorescence staining and cell counting of Olig2 **(D,E)**, CNPase **(D,F)**, DCX **(D,G)**, and GFAP **(D,H)** on AGC1^+/+^ and AGC1^±^ 7DIV spontaneously differentiated neurospheres following HAT inhibition with curcumin 5 and 10 μM. Values are mean ± SD of 3 different fields acquired for each condition. 60X objective; 50 μm bar scale. WB and relative densitometries of panAcH3 **(I,J),** Olig2 **(I,K)**, CNPase **(I,L)**, DCX **(I,M)**, and GFAP **(I,N)** expression in AGC1^+/+^ and AGC1^±^ differentiated neurospheres following HAT inhibition with curcumin 5 and 10 μM. Values are mean ± SD of 3 independent experiments. ^#^*p* < 0.05, ^##^*p* < 0.01, ^###^*p* < 0.001, compared to DMSO control, respectively; *p < 0.05, **p < 0.01, ***p < 0.001, compared to each treated control; two-way ANOVA (Bonferroni’s *post hoc* test).

Similar treatments have been performed on neurospheres from the SVZ of AGC1^±^ and AGC1^+/+^ mice. Neurospheres have been allowed to grow in culture for 7 days with 5 or 10 μM curcumin (based on MTT assay, not shown) and their number and diameter have been evaluated (Gil-Perotín et al., 2013). As already demonstrated, AGC1^±^ neurospheres appeared smaller, but in greater number compared to AGC1^+/+^ ones (DMSO cells) (Petralla et al., 2019). Curcumin treatments induced a significant reduction in neurospheres diameter in both AGC1^±^ and AGC1^+/+^ cells. However, the treatment increased the number of AGC1^+/+^ neurospheres, while it decreased the number of AGC1^±^ ones, probably due to an earlier arrest in the proliferation as a consequence of their lower intrinsic proliferation rate (Figures 8A–C).

To further study the effect of HAT inhibition on NPCs spontaneous differentiation, AGC1^±^ and AGC1^+/+^ neurospheres have been plated on fibronectin and exposed to 5 or 10 μM curcumin for 7 days. Fluorescence quantification after staining with Olig2 (oligodendrocyte lineage marker), CNPase (myelinating oligodendrocytes marker), DCX (neural precursor cells marker; Francis et al., 1999), and GFAP (astrocytes marker; Nolte et al., 2001) confirmed AGC1^±^ neurospheres being characterized by reduced Olig2^+^ cells and increased CNPase^+^, DCX^+^, and GFAP^+^ cells compared to AGC1^+/+^ neurospheres (Figures 8D–H; Petralla et al., 2019). Curcumin treatment-induced almost no change in AGC1^+/+^ neurospheres differentiation, besides a little, but significant reduction in GFAP^+^ cells after 10 μM treatment (Figure 8H). Conversely, it leads to a significant reduction in CNPase^+^ (Figures 8D,F) and GFAP^+^ (Figures 8D,H) cells in AGC1^±^ neurospheres, with no relevant changes in both Olig2^+^ (Figures 8D,E) and DCX^+^ (Figures 8D,G) cells. Further confirmation has been obtained by WB analysis (Figures 8I–N). As previously shown, in DMSO-treated controls, the Olig2 expression turned out lower, while CNPase, GFAP, and even DCX resulted in a significant increase in AGC1^±^ than in AGC1^+/+^ neurospheres. Curcumin treatment has no significant effect on Olig2 and DCX expression in both AGC1^±^ and AGC1^+/+^ neurospheres, but it significantly decreases both CNPase and GFAP expression in AGC1^±^ neurospheres.

Altogether, these data demonstrate that HATs inhibition arrests proliferation and stimulates differentiation of immortalized OPCs toward myelinating oligodendrocytes. This pro-differentiating effect is more evident in control cells than after AGC1 downregulation, thus creating a condition similar to AGC1 deficiency. In NPCs represented by neurospheres, AGC1 silencing induces the differentiation toward both differentiated glial and neuronal cells. HATs inhibition has almost no effect on AGC1^+/+^ neurospheres, while it reduces the differentiation toward both oligodendrocytes and astrocytes, with no effect on OPCs and the commitment toward neurons in AGC1^±^ cells.

### Histone Deacetylases Inhibition Through Suberanilohydroxamic Acid in the Proliferation/Differentiation of Oligodendrocytes Precursor Cells and Neuronal Precursor Cells

The role of HDACs in the proliferation/differentiation of OPCs and NPCs, as well as their alteration in AGC1 deficiency, was investigated in Oli-Neu cells and neurospheres treated with SAHA, a broad-spectrum HDAC inhibitor suppressing the family members in multiple HDAC classes (Xu et al., 2007) and approved by FDA for cancer therapy. In cells, SAHA is known to be involved in several processes i.e., it induces the accumulation of acetylated histones and acetylated non-histone proteins in transcription factor TF complexes (e.g., TFIIB), which alter gene expression; it promotes acetylation of proteins regulating cell proliferation (e.g., Rb), protein stability (e.g., Hsp90), apoptosis (e.g., Bcl-2 family of proteins), cell motility (e.g., tubulin), and angiogenesis (HIF-1α); it alters the expression of proteins (e.g., Trx), which modulate the accumulation of reactive oxygen species (ROS) that facilitated cell death (Johnstone and Licht, 2003; Rosato et al., 2003; Shao et al., 2004; Bhalla, 2005; Dokmanovic and Marks, 2005; Minucci and Pelicci, 2006; Yoo and Jones, 2006).

**FIGURE 9:**
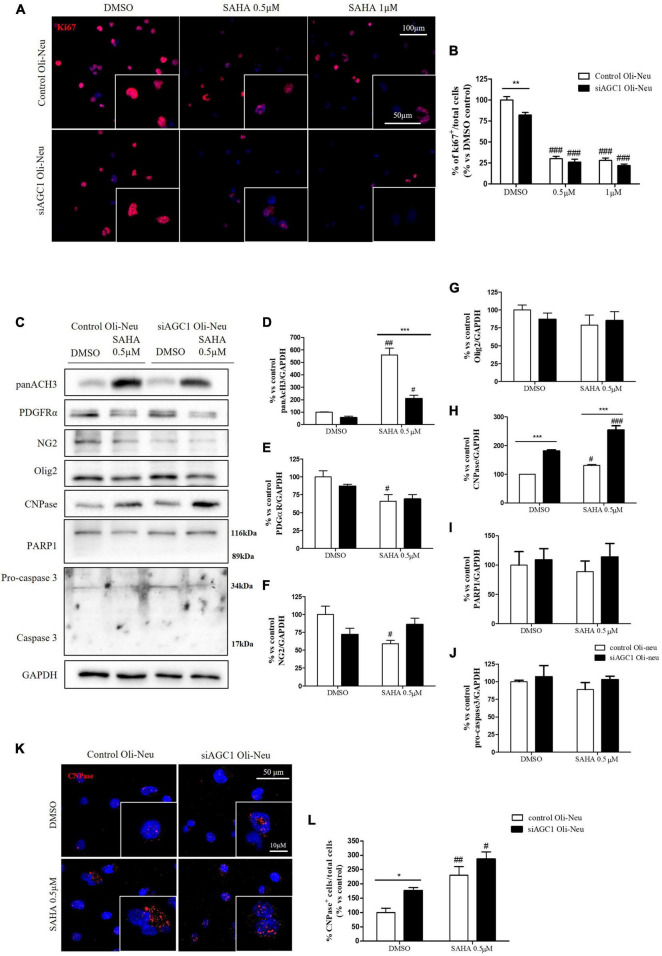
Suberanilohydroxamic acid (SAHA)-mediated HDACs inhibition differentially reduces proliferation and promotes differentiation in control and AGC1-silenced Oli-Neu cells. Immunofluorescence staining of Ki67 proliferation marker **(A)** and Ki67^+^ cell count analysis **(B)** in Oli-Neu cells after 24 h treatment with 0.5 or 1.0 μM SAHA. Values are expressed as the *ratio* of Ki67^+^ cells (red)/total cells; nuclei were labeled with DAPI. Values are mean ± SD of 3 independent experiments; 3 different fields were acquired for each condition. 40X objective; 50 and 100 μm bar scale. WB and relative densitometries of panAcH3 **(C,D)**, PDGFRα **(C,E)**, NG2 **(C,F)**, Olig2 **(C,G)**, CNPase **(C,H)**, PARP1 **(C,I)**, and pro-caspase3 **(C,J)** expression in OliNeu cells after 48 h treatment with 0.5 μM SAHA. Immunofluorescence staining of CNPase **(K)** and CNPase^+^ cell count analysis **(L)** in OliNeu cells after 48 h treatment with 0.5 μM SAHA. Values are expressed as the *ratio* of CNPase^+^ cells (red)/total cells; nuclei were labeled with DAPI. Three different fields were acquired for each condition. Values are mean ± SD of at least 3 independent experiments. 40X objective; 50 and 10 μm bar scale. ^#^*p* < 0.05, ^##^*p* < 0.01, ^###^*p* < 0.001, compared to DMSO control, respectively; *p < 0.05, **p < 0.01, ***p < 0.001, compared to each treated control; two-way ANOVA (Bonferroni’s *post hoc* test).

**FIGURE 10:**
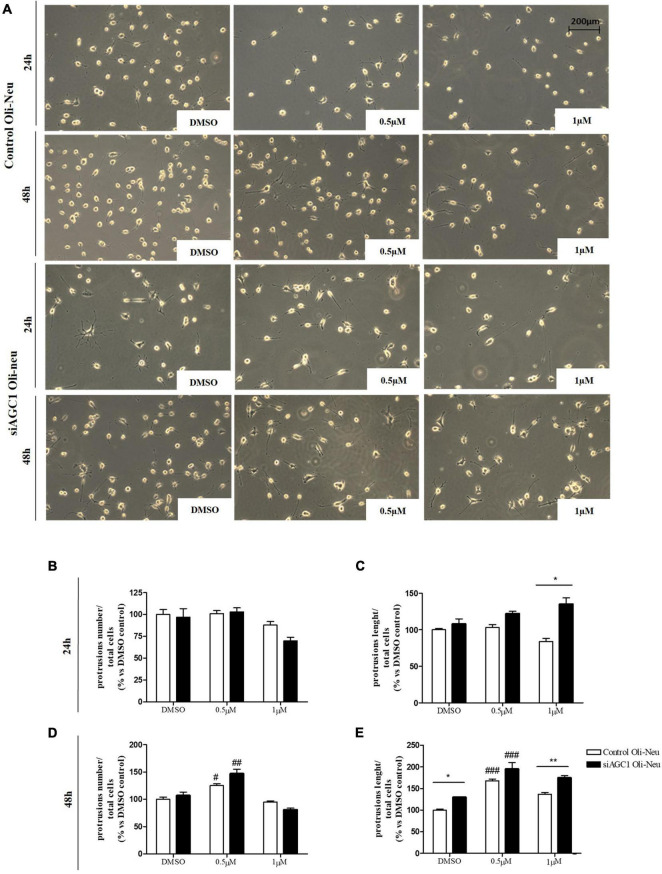
Histone deacetylases inhibition leads to Oli-Neu cells differentiation with a decrease in filament number and increases in filaments length in siAGC1 cells compared to control ones after 48 h SAHA treatment. Optical microscope proliferation/differentiation analysis in siAGC1 and control Oli-Neu cells following 24 and 48 h of SAHA treatment **(A)**. The number and lengths of filaments were counted and measured, respectively, and analyses were carried out with Fuji Imagej2 software **(B–E)**. Analysis was carried out with Fuji Imagej2 software. Values are mean ± SD of 3 independent experiments; 3 different fields were acquired for each condition. 20X objective; 200 μM bar scale. ^#^*p* < 0.05, ^##^*p* < 0.01, ^###^*p* < 0.001, compared to DMSO control, respectively; *p < 0.05, **p < 0.01, compared to each treated control; two-way ANOVA (Bonferroni’s *post hoc* test).

Control and siAGC1 Oli-Neu cells have been treated with SAHA 0.5 and 1.0 μM for 24 h. Counting of Ki67^+^ cells showed lower proliferation in siAGC1 cells compared to the control (DMSO-treated cells), whereas SAHA treatment significantly reduced proliferation in both the cell lines, at both concentrations (Figures 9A,B). The WB analysis confirmed a strong induction of histone H3 acetylation following HDACs inhibition, which is more evident in the control Oli-Neu cells than in siAGC1 ones (Figures 9C,D). PDGFαR, Olig2, and NG2, as expected, were less expressed in siAGC1 Oli-Neu cells, but only PDGFαR and NG2 significantly decreased after SAHA treatment only in the control cells, indicating a decrease in the OPCs pool (Figures 9C,E–G). Conversely, CNPase expression significantly increased in both cell lines following HDACs inhibition (Figures 9C–H), as confirmed in immunofluorescence (Figures 9K,L), to suggest a stimulated differentiation in OPCs when HDACs is inhibited. Additionally, through WB analysis, no differences were observed for the pro-apoptotic marker, caspase3 (both precursor and cleaved versions of the enzyme) (Figures 9C,J), the full length PARP1 (116 kDa), and the large fragment (89 kDa) of PARP1 resulting from caspase cleavage (Figures 9C,I), and the anti-apoptotic protein, BCL-2 (data not shown) in treated vs. control cells, excluding apoptosis and cell death in the reduction of OPCs pool. From microscopic morphology analysis, protrusion length, but not the protrusion number, significantly increased in siAGC1 Oli-Neu cells after 24 h with 1.0 μM SAHA (Figures 10A–C). However, at 48 h, greater effects were obtained from both cell lines with 0.5 μM SAHA, where protrusion number and length turned out significantly higher compared to DMSO-treated controls, respectively (Figures 10A,D,E). These data demonstrated that the inhibition of HDACs, and the consequent increase of histone H3 acetylation, limits the proliferation of OPCs with a parallel increase in their differentiation, being this effect more evident in control cells, where it leads to a condition similar to AGC1 deficiency.

**FIGURE 11:**
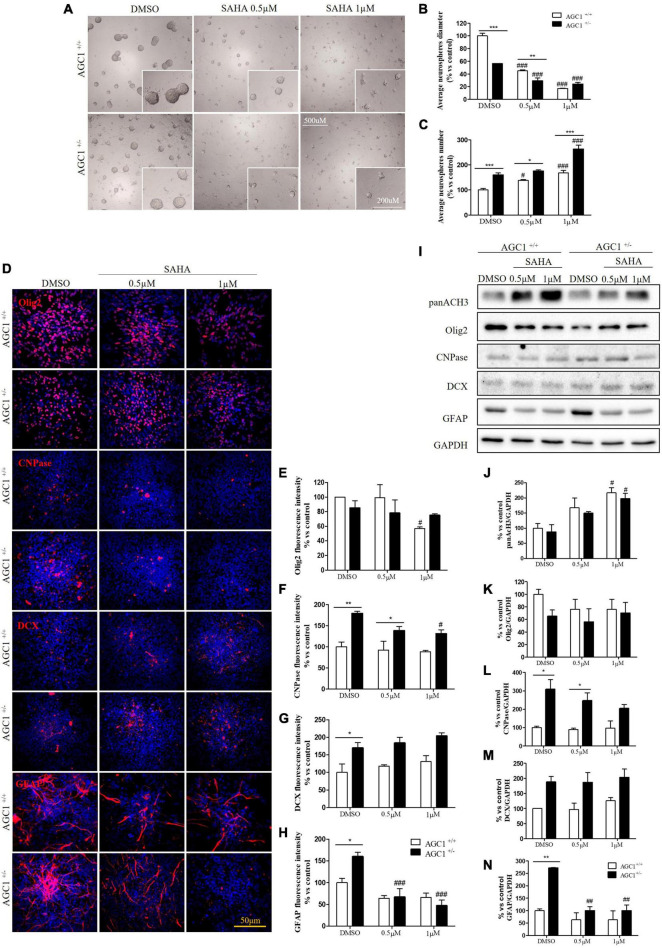
Suberanilohydroxamic acid-mediated HDACs inhibition affects proliferation and commitment through glial cells in spontaneously differentiated AGC1^+/+^ and AGC1^±^ neurospheres. Optical microscope images **(A)** and counting of number **(B)** and diameter **(C)** of neurospheres after 7 days of culture in presence of SAHA 0.5 and 1 μM. Average number and size were measured with Fuji Imagej2 software using an automated colony and cell counting method; only aggregates bigger than 400 μm^2^ were considered. Values are mean ± SD of 5 different fields acquired for each condition. 10X objective; 500 and 200 μm bar scale. Immunofluorescence staining and cell counting of Olig2 **(D,E)**, CNPase **(D,F)**, DCX **(D,G)**, and GFAP **(D,H)** on AGC1^+/+^ and AGC1^±^ 7DIV spontaneously differentiated neurospheres following HDACs inhibition with SAHA 0.5 and 1 μM. Values are mean ± SD of 3 different fields acquired for each condition. 60X objective; 50 μm bar scale. WB and relative densitometries of panAcH3 **(I,J)**, Olig2 **(I,K)**, CNPase **(I,L)**, DCX **(I,M)**, and GFAP **(I,N)** expression in AGC1^+/+^ and AGC1^±^ differentiated neurospheres following HDAC inhibition with SAHA 0.5 and 1 μM. Values are mean ± D of 3 independent experiments. ^#^*p* < 0.05, ^##^*p* < 0.01, ^###^*p* < 0.001, compared to DMSO control, respectively; *p < 0.05, **p < 0.01, ***p < 0.001, compared to each treated control; two-way ANOVA (Bonferroni’s *post hoc* test).

Suberanilohydroxamic acid treatment has been also evaluated on the proliferation/differentiation of AGC1^±^ and AGC1^+/+^ neurospheres. AGC1^+/+^ neurospheres treated with the culture of SAHA of 0.5 or 1.0 μM (DMSO as control) for 5 days (not 7 days as for curcumin, because of its toxicity in long-time culture; Zhou et al., 2011), revealed a significant decrease in average diameter, with a parallel increase in number. In the same experimental conditions, DMSO-treated AGC1^±^ neurospheres confirmed their higher number and smaller size compared to AGC1^+/+^ controls, and after SAHA treatment, showed a further significant increase in number and decrease in size (Figures 11A–C). Therefore, pharmacological HDACs inhibition seems to determine a reduction in the proliferation of NPCs in neurospheres with a more limited effect on AGC1^±^ ones compared to AGC1^+/+^, probably due to the pathological reduction in HDACs expression and histone acetylation.

The effect of the inhibition of HDACs by SAHA on the differentiation of NPCs has been then tested on both AGC1^±^ and AGC1^+/+^ neurospheres spontaneously differentiated on fibronectin. Unlike AGC1^+/+^, the fewer number of Olig2^+^ cells in AGC1^±^ neurospheres were not further reduced by SAHA treatments (Figures 11D,E), whereas the higher number of CNPase^+^ cells significantly decreased, with no change in AGC1^+/+^ control (Figures 11D,F). Neuronal-restricted progenitors, which express DCX as a specific marker and are more present in AGC1^±^ neurospheres compared to AGC1^+/+^ ones (Petralla et al., 2019), did not reveal any change (Figures 11D,G). Astrocytes, however, were identified as GFAP^+^ cells and significantly more expressed in AGC1^±^ than in AGC1^+/+^ neurospheres^+/+^ (Petralla et al., 2019), showed a strong reduction in treated AGC1^±^ but not in the control cells (Figures 11D,H).

Western Blot analysis confirmed a significant increase in histone H3 acetylation in NPCs following SAHA treatment (Figures 11I,J). As observed through immunostaining, while SAHA did not change Olig2 and DCX expression, the former lower (Figures 11I,K) and the latter higher (Figures 11I,M) in AGC1^±^ neurospheres as compared to AGC1^+/+^, respectively, the pharmacological HDACs inhibition determined a significant reduction in CNPase (Figures 11I,L) and GFAP (Figures 11I,N) expression in AGC1^±^ neurospheres only, where these markers have a basal significant higher level compared to control conditions, as previously demonstrated (Petralla et al., 2019). Therefore, the inhibition of HDACs has only a limited effect on the proliferation of OPCs, while it significantly induces the differentiation of OPCs, both in cells with downregulated AGC1 and in controls, being this effect more evident in the latter ones. The SAHA effect on the proliferation of OPCs has been observed in both Oli-Neu cells and neurospheres. On the contrary, in AGC1^±^ neurospheres, in which the number of oligodendrocytes and astrocytes is significantly higher than in AGC1^+/+^ cells, the inhibition of HDACs determines a reduction in both differentiated glial cells number, with no effects on the neural progenitors.

## Discussion

The deficiency of AGC1 is a mitochondrial disorder manifesting with developmental epileptic encephalopathy, recently defined as a leukodystrophy (Kavanaugh et al., 2019). These childhood white matter disorders (WMDs) display neurologic features, such as motor deficits, hypotonia, and epileptic seizures associated with important systemic symptoms, and involve a wide range of heterogeneous genetic and metabolic disorders, also including mitochondrial encephalopathies (Ashrafi and Tavasoli, 2017; Ashrafi et al., 2020). This is the reason why the study of the ultra-rare, genetic AGC1 deficiency can provide useful information to understand the pathogenic mechanisms at the basis of the wide family of leukodystrophies. Besides, WMDs, leukodystrophy, and leukoencephalopathy have been recently involved in dementia (Lok and Kwok, 2021), further increasing the interest in understanding the molecular mechanisms underlying these disorders.

White matter is composed of myelinated neuronal axons and glial cells, mainly OPCs and oligodendrocytes, but astrocytes and microglia are also present. Myelination and remyelination are the main processes that modulate the correct functioning of the white matter, both during development and in adulthood. In CNS, myelin is formed by oligodendrocytes that wrap axons through multiple concentric membranous layers. Oligodendrocytes, derived from the maturation of OPCs, are highly migratory and are actively proliferative glial progenitors, representing about 5% of mouse brain cells. OPCs in turn originates from the gliogenic commitment of NPCs, mostly localized in the SVZ (Goldman and Kuypers, 2015). The NPCs can give rise to different cells in CNS: neurons, astrocytes, and oligodendrocytes, both during brain development and in adulthood (Gage, 2000). Formerly published data from our lab have previously shown a deficit in the proliferation of OPCs with low AGC1 expression (siAGC1 Oli-Neu cells), with no change in their differentiation into oligodendrocytes. This proliferation defect correlated with the dysregulation in the expression of growth factors involved in the proliferation/differentiation of OPCs and these data were confirmed in AGC1^±^ mice. Furthermore, in the neurospheres from SVZ of the murine model, we observed a reduced number of OPCs with a parallel increase in oligodendrocytes, astrocytes, and precursor neurons, as well as an imbalance in the proliferation/differentiation pathways (Petralla et al., 2019). It is noteworthy that in our model of OPCs with downregulated AGC1, we do not observe any mitochondrial respiration deficit, thus suggesting that the alterations in the proliferation/differentiation pathways may be not directly due to a bioenergetic dysfunction, but due to a different molecular mechanism (Petralla et al., 2019).

It is widely recognized that transcriptional (Meijer et al., 2012; Yan et al., 2013; Magri et al., 2014; Palazuelos et al., 2014; Song et al., 2015; Carroll et al., 2018) and epigenetic mechanisms, especially histone acetylation (Juliandi et al., 2010; Emery and Lu, 2015; Hernandez and Casaccia, 2015; Tiane et al., 2019; Zhang et al., 2020), are major contributors to OPCs and NPCs proliferation and differentiation and that their dysregulation is involved in demyelinating disorders (Hsieh and Gage, 2004; Podobinska et al., 2017). Acetylation and deacetylation of histones are highly dynamic processes that depend on the activity of two groups of enzymes: HATs and HDACs. HDAC1, 2, 3, and 4 are the most relevant isoforms in CNS (D’Mello, 2020), while, among the HATs, CBP is mainly active in CNS. The CBP, by binding with the transcription factor CREB (Sheikh, 2014; Lipinski et al., 2019), creates a direct link between transcriptional and epigenetic regulation of gene expression in the development, differentiation, and function of the brain cells (Bito and Takemoto-Kimura, 2003). CREB, which is activated by its phosphorylation, is known to be involved in neuronal differentiation and survival, as well as in many other brain functions (Yan et al., 2013).

In both the investigated *in vitro* models of AGC1 deficiency, we observed a strong reduction in the expression of the transcription factors, Olig2 and Myc, which can be related to the reduction in the proliferation of OPCs. Instead, CREB is increased in siAGC1 Oli-Neu cells and reduced in AGC1^±^ neurospheres, compared to the controls, experimental evidence that could be explained by the multicellular composition of the NPCs model, in which we previously demonstrated a shift in the spontaneous differentiation of both the neuronal and glial cells (Petralla et al., 2019). Moreover, the altered expression pattern of the TFs, investigated in this study, is accompanied by changes of HATs enzyme and in particular of CBP, found significantly reduced in both OPCs and NPCs with downregulated AGC1. As a consequence, histone acetylation is reduced, and the parallel reduction in HDACs activity and expression appears not sufficient to sustain the necessary histone acetylation and in turn the proliferation of our models of AGC1 deficiency.

The role of histone acetylation/deacetylation in the proliferation deficit of OPCs and NPCs with low AGC1 was then investigated in the presence of the HATs inhibitor, curcumin (Balasubramanyam et al., 2004; Sunagawa et al., 2018), and the HDACs inhibitor, SAHA (Xu et al., 2007; Zhou et al., 2011). Curcumin, by decreasing histone acetylation, reduces the proliferation of OPCs in the control and stimulates their differentiation toward oligodendrocytes, hence mimicking the condition described in siAGC1 Oli-Neu cells. Furthermore, curcumin has greater effects also on NPCs commitment, where it reduces the gliogenic differentiation toward both astrocytes and oligodendrocytes, without changing neurogenesis and the proliferation of OPCs. The data here presented, therefore, suggest that the lower CBP level and in turn HATs activity in OPCs with impaired AGC1 may cause their proliferation deficit, as well as the precocious differentiation of OPCs to mature oligodendrocytes, whereas, in NPCs, it determines an alteration in the commitment toward glial cells and limited effect on NPCs neuronal commitment, in line with what was previously hypothesized (Hernandez and Casaccia, 2015).

**FIGURE 12:**
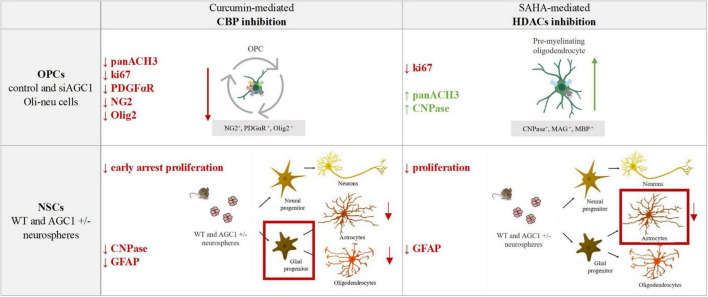
Summary table of increased/decreased markers involved in OPCs and NSCs proliferation/differentiation following curcumin-mediated CBP inhibition and SAHA-mediated HDACs inhibition in control and siAGC1 Oli-neu and WT and AGC1^±^ neurospheres.

The pharmacological inhibition of HDACs by SAHA has only a limited effect on the proliferation of OPCs, inducing a slight differentiation toward oligodendrocytes, especially in the control cells, whereas in NPCs, it produces a strong reduction in differentiated astrocytes with almost no effect on neural progenitors (Figure 12).

Overall, our data showed that changes in HATs and HDACs activity and consequently in histone PTMs may underly the altered balance between proliferation and differentiation in the investigated models with downregulated AGC1. On the other hand, and more in general, our data pointed out that pharmacological inhibition or pathologic conditions impairing histone acetylation could be responsible for the proliferation deficit of brain progenitors with HATs likely more involved in the gliogenic commitment of NPCs and the proliferation/differentiation balance of OPCs, while HDACs may play a later role in the differentiation of glial precursor cells toward astrocytes.

An important issue arising from the data presented here shows the role of mitochondria, and in particular of AGC1, for the generation of acetyl groups. Histone acetylation by HATs requires acetyl-CoA produced by mitochondrial activity, and cellular levels of this metabolite depend on energy status. The AGC1 regulates and preserves mitochondrial pyruvate oxidation through the malate/aspartate, NADH shuttle (Kerkhofs, 2020), a biochemical pathway essential to supply the energy demands of the brain cells by feeding the tricarboxylic acid (TCA) cycle. In addition, the import of pyruvate in the mitochondrial matrix allows for the synthesis of citrate that releases acetyl-CoA *via* citrate lyase once exported to the cytosol. Therefore, AGC1 function may be determinant for histone acetylation, thus representing an important link between mitochondrial metabolism and gene expression (Menzies et al., 2016; Matilainen et al., 2017). It has been previously demonstrated that chronic mitochondrial dysfunction leads to a reduced mitochondrial output of acetyl-CoA, which in turn limits the HATs activity and histone acetylation, overall contributing to the regulation of gene expression in the nucleus (Lozoya et al., 2019; Santos, 2021). This evidence resembles what we have observed in our models of BPCs with AGC1 deficiency. Indeed, our data revealed no significant reduction of mitochondrial respiration in the OPCs model with low AGC1. We can speculate that the residual activity of the carrier is sufficient to sustain the mitochondrial pyruvate oxidation and in turn OXPHOS (Llorente-Folch et al., 2013; Szibor et al., 2020). However, AGC1 silencing may limit the amount of the citrate synthesized in the TCA cycle that is exported in the cytosol, hence providing less acetyl-CoA for the whole OPCs and in turn affecting the histone acetylation. It should also be considered that a further important physiological source of acetyl groups in OPCs is the NAA synthesized in neurons and transaxonally transported to oligodendrocytes (Wibom et al., 2009) for myelin synthesis. In control OPCs here investigated, the AGC1 may compensate for the absence of the neuronal-derived NAA with minor consequences in histone acetylation than in OPCs with silenced AGC1. Whether exogenous NAA may have a role in the proliferation/differentiation of OPCs will deserve further experimentation.

Overall, the data presented in this study suggested a new role for the mitochondrial aspartate-glutamate carrier that links metabolism, epigenetics, and gene expression regulation in the brain precursor cells (BPCs). This connection is particularly remarkable if we take into consideration, the positive effect produced in patients with AGC1 deficiency in which NAA levels and myelination significantly increase (Dahlin et al., 2015; Pérez-Liébana et al., 2020; Broeks et al., 2021). This nutritional intervention is based on a high fat, low-carbohydrate diet that uses lipids and ketone bodies (β-hydroxybutyrate and acetoacetate), rather than glucose, as primary fuels, thus inducing favorable metabolic adaptions. KD is effectively utilized as a metabolic treatment in a wide range of neurological metabolic diseases, as it is neuroprotective and strongly improves myelination in drug-refractory epilepsy, mitochondrial diseases, leukodystrophies, and multiple sclerosis (Finsterer, 2011; Stumpf et al., 2019; Bahr et al., 2020; Norwitz et al., 2020; Rudy et al., 2020). KD may alternatively fulfill the energetic demands of the cells, but its beneficial effects could also be attributed to epigenetic mechanisms, that might be consequential to the increased intracellular acetyl-CoA pool formed by the administration of ketone bodies and to the debated HDACs inhibitory potential of β-hydroxybutyrate, both sustaining histone acetylation (Chriett et al., 2019; Dąbek et al., 2020). The pro-acetylating effect of KD could also improve the proliferation of BPCs, thus providing cells that can differentiate between oligodendrocytes and support myelination.

Similar to AGC1 deficiency, dysfunctions in OPCs and NSCs are now widely recognized in many neuropathologies. The OPCs have been involved in demyelinating conditions, where these cells are the main source of regenerating oligodendrocytes and the inadequate expansion of the OPCs pool may be a limiting factor for successful remyelination.

Oligodendrocyte precursor cells may also change in response to acute injuries, such as ischemia and trauma, and white matter abnormalities with OPCs changes have been observed in neuropsychiatric disorders, including depression and schizophrenia (Nishiyama et al., 2021a).

In conclusion, our study in models of the ultra-rare genetic disease, AGC1 deficiency provided new information to describe the pathogenetic mechanisms of the disease that could also be useful to clarify the role of histone acetylation in the regulation of gene expression in the BPCs in other neuropathologies affecting white matter. The comprehension of the molecular pathways and epigenetic mechanisms regulating proliferation and/or differentiation of OPCs could lead to the identification of new targets for potential therapeutic approaches acting on histone-modifying enzymes may increase the OPCs pool and adequately sustain their differentiation toward oligodendrocytes for correct myelination/remyelination processes (Monti, 2021) in neurodegenerative, neuropsychiatric, and neurodevelopmental diseases (Ganguly and Seth, 2018; D’Mello, 2020; Gupta et al., 2020).

## Data Availability Statement

The raw data supporting the conclusions of this article will be made available by the authors, without undue reservation.

## Ethics Statement

The animal study was reviewed and approved by the University of Bologna Bioethical Committee (Protocol no 3/79/2014).

## Author Contributions

BM and FL conceptualized the general approach and designed the experiments. EP, SP, GB, BR, LC, MCM, SB, MM, FD, and FM performed the experiments. EP, BM, and FL analyzed and interpreted the data, as well as drafted the manuscript. LV, LP, and MV helped design the experiments and contributed methodology. All authors reviewed and approved the final version of the manuscript.

## Conflict of Interest

The authors declare that the research was conducted in the absence of any commercial or financial relationships that could be construed as a potential conflict of interest.

## Publisher’s Note

All claims expressed in this article are solely those of the authors and do not necessarily represent those of their affiliated organizations, or those of the publisher, the editors and the reviewers. Any product that may be evaluated in this article, or claim that may be made by its manufacturer, is not guaranteed or endorsed by the publisher.
